# Volatiles Profile of the Floral Organs of a New Hybrid *Cymbidium*, ‘Sunny Bell’ Using Headspace Solid-Phase Microextraction Gas Chromatography-Mass Spectrometry Analysis

**DOI:** 10.3390/plants8080251

**Published:** 2019-07-27

**Authors:** Yun-Su Baek, Mummadireddy Ramya, Hye-Ryun An, Pil-Man Park, Su-Young Lee, Nam-In Baek, Pue-Hee Park

**Affiliations:** 1 Floriculture Research Division, National Institute of Horticultural and Herbal Science, RDA, Wanju 55365, Korea; 2 Agency for Korea National Food Cluster (AnFC), Foodpolis110, Dongchonje-gil, Wanggung-myeon, Iksan-si, Jeollabuk-do 54576, Korea; 3 Graduate School of Biotechnology and Department of Oriental Medicine Biotechnology, Kyung Hee University, Yongin 17104, Korea; 4 Department of Horticultural Science and Biotechnology, Seoul National University (SNU), Seoul 08826, Korea

**Keywords:** *Cymbidium* hybrid, floral scents, linalool, monoterpenes, orchids, volatile compounds

## Abstract

*Cymbidium* is one of the most important genera of flowering plants in the Orchidaceae family, and comprises a wide variety of beautiful and colorful species. Among these, only a few species possess floral scents and flavors. In order to increase the availability of a new *Cymbidum* hybrid, “Sunny Bell”, this study investigated the volatile floral scents. Volatiles of the floral organs of the new *Cymbidium* hybrid, “Sunny Bell”, at the full-flowering stage were characterized with headspace solid-phase microextraction gas chromatography-mass spectrometry (HS-SPME-GC-MS) analysis. A divinylbenzene-carboxen-polydimethylsiloxane (DVB-CAR-PDMS) fiber gave the best extraction for volatile components. Twenty-three components were identified as the main volatiles for the floral organs of the new *Cymbidium* hybrid, “Sunny Bell” at the full-flowering stage; twelve compounds in the column, sixteen compounds in the labellum, eleven compounds in the sepals, and nine compounds in the petals were identified. Terpenes are the major source of floral scents in this plant. As a result of GC-MS analysis, the most abundant compound was linalool (69–80%) followed by α-pinene (3–27%), 4,8-dimethyl-1,3,7-nonatriene (5–18%), eucalyptol (6–16%), and 2,6-dimethylnonane (2–16%). The main components were identified as monoterpenes in the petals and sepals, and as monoterpenes and aliphatics in the column and labellum. The results of this study provide a basis for breeding *Cymbidium* cultivars which exhibit desirable floral scents.

## 1. Introduction

Orchids, a flowering plant with over 800 described genera and 25,000 species, are prized for their beauty and long flowering, exhibiting flowers of an incredible variety of size, shape, and color [1]. *Cymbidium* comprises approximately 44 species that are native to the Himalayas, tropical regions of Southeast Asia, and Australia [2]. It has been reported that *Cymbidium* flowers are rich in volatile compounds including cineole, isoeugenol, and (-)-α-selinene [3]. Floral scent and color are major traits for floriculture crops in developing new cultivars of *Cymbidium*. Therefore, headspace solid-phase microextraction gas chromatography-mass spectrometry (HS-SPME-GC-MS) was used to characterize the floral scent of the new *Cymbidium* hybrid, “Sunny Bell”.

**Figure 1:**
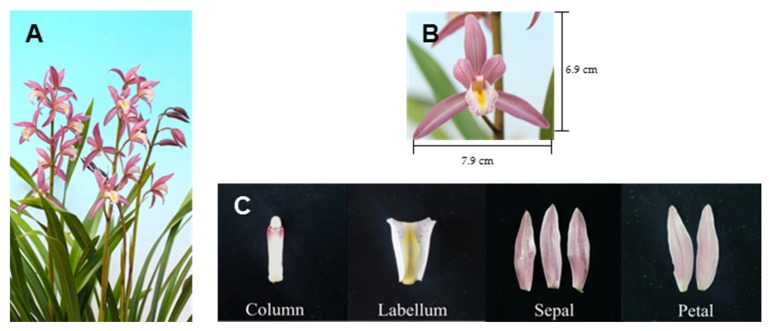
New hybrid *Cymbidium* “Sunny Bell” plant (**A**), flower (**B**), and floral organs (**C**).

**Figure 2:**
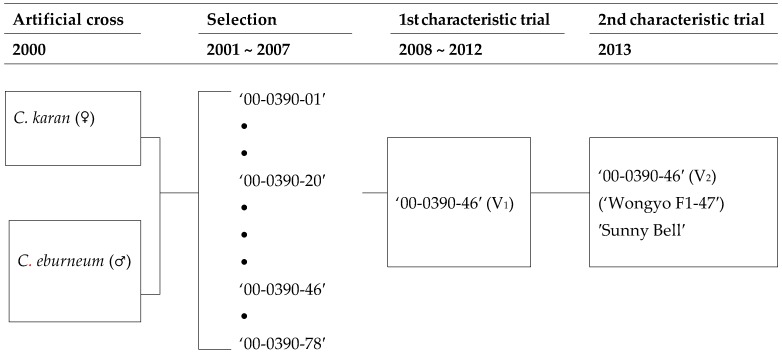
Pedigree diagram of the new hybrid, *Cymbidium* “Sunny Bell”. “•” indicates the number of plants in each lane.

A *Cymbidium* variety, “Sunny Bell” (*Cymbidium karan* × *Cymbidium eburneum*) was developed at the National Institute of Horticultural and Herbal Science, Rural Development Administration, Suwon, Korea in 2013 (Figure 1). Different spider-type cultivars showed differences in the quantity and quality of floral aroma volatiles. Different accessions of *C.* also showed differences in the amounts of floral scent volatiles. This hybrid came from the progenies crossed between *C. eburneum* and C. *karan* in 2000 (Figure 2). 

Finally, 101 seedlings were obtained after planting and acclimatization in a green house. In 2007, one line was selected based on its performance characteristics including flower color, leaf shape, flower stalk, and growth rate, and named “Wongyuo F1-47”. A proceeding line coded 000390-46 possessed uniformity and excellent characteristics with a floral scent. The line 00-0390-46 has a longer flowering period because of its relatively larger diameter and greater number of flowers than 00-0390-20, which is a comparable variety; therefore, consumer preference for this cultivar is stronger than that for others. The line selected after the second analysis of plant characteristics was named “Sunny Bell”. This hybrid had light purple-colored petals and lips (RP59B) and large-sized flowers (diameter 7.9 cm). The general impression of the petals and sepals showed an incurved shape. The flowers started blooming from in February under optimal culture conditions. “Sunny Bell” had about 7–8 flowers per stalk, with large-sized plants. The peduncle attitude was erect (Table 1 and Table 2).

Headspace analysis can be used to determine the composition of natural materials and to provide broad olfactory profiles [4]. Solid phase microextraction (SPME) is a simple, fast, sensitive, and convenient sample preparation technique that minimizes solvent usage while integrating sampling and sample preparation steps prior to instrumental analysis [5]. Recently, SPME has been widely applied to the sampling and analysis of aromatic and volatile biological pharmaceutical samples. For example, the authors of [6] reported forty-three compounds in the flower of *Vicia sativa* L. and the authors of [7] analyzed and identified volatile constituents which included alcohols, aldehydes, esters, acids, ketones, terpenes, C13-norisoprenoides, and sulfur compounds from two species of the *Brassicaceae* (Crucifer) family using HS-SPME-GC-MS [6,7]. Solid-phase microextraction (SPME) is a new type of sample pretreatment technology that allows the rapid and simple extraction of small amounts of volatile compounds. This technique has high reproducibility under the same test conditions and is suitable for floral scent analysis. In this investigation, we performed headspace solid-phase microextraction (HS-SPME) followed by gas chromatography-mass spectrometry (GC-MS) to analyze the floral scent volatiles. Nowadays, several types of SPME fiber coatings are available for the extraction of analytics. Among them, non-polar polydimethylsiloxane (PDMS) fibers are preferred for the extraction of non-polar analytes, including many volatile flavor compounds. Carboxen-polydimethylsiloxane (CAR-PDMS) fibers exhibit better extraction efficiency than 50/30 μm DVB/CAR/PDMS coated SPME fiber attached to a manual PDMS fiber, and similar fibers, but show inferior repeatability and their equilibration is more time-consuming [8].

Monoterpenes, sesquiterpenes, and aliphatics have been identified as the major volatile compounds in *Cymbidium*, with their total exceeding 90%. While there have been previous reports on the analysis of volatile components [(*E*)-4-hexadecen-6-yne, 6-oxoheptanoic acid methyl ester, dodecane hexadecanoic acid, isopropyl myristate, and tetradecanoic acid] of *Cymbidium* varieties [9,10], there are no reports on the characteristics of the volatiles contributing to the floral scents of the new “Sunny Bell” hybrid. Thus, in the present study, HS-SPME coupled with GC-MS was used to characterize the volatile components of “Sunny Bell” flowers. Specifically, this study aimed to evaluate the volatile polymorphisms of different floral organs from “Sunny Bell” to determine the floral organs of significant scent.

## 2. Results and Discussion

The floral volatile components of the “Sunny Bell” *Cymbidium* hybrid were analyzed using HS-SPME coupled with GC-MS. Each peak was identified by matching their spectra with those recorded in the National Institute of Standards and Technology (NIST) 14 mass spectral library and published data, as well as analysis of the Retention indices [RI] or Gas Chromatography the retention time [GC r.t.] data, and confirmed through analysis of the fragmentation pattern in mass spectra. Table 3 shows the 23 volatile components identified as the volatiles of the “Sunny bell” flowers; of which 94.05% were in the column, 97.23% in the labellum, 98.82% in the sepals, 99.80% in the petals, and 88.95% in the whole flower (new hybrid *Cymbidium*, “Sunny Bell”). These volatiles were grouped based on biochemical synthesis pathways [11].

**Figure 3:**
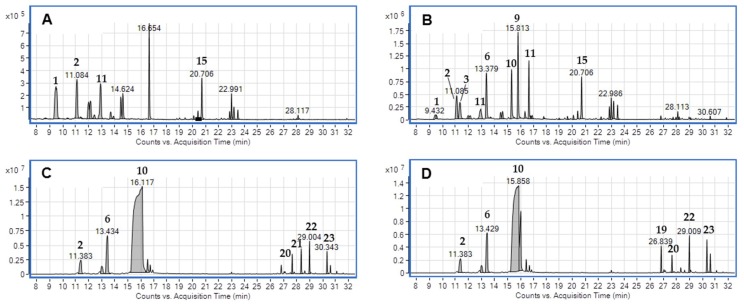
Representative total ion chromatograms for column (**A**), labellum (**B**), sepals (**C**), and petals (**D**) of the new hybrid, *Cymbidium* “Sunny Bell” flower analyzed by HS-SPME-GC-MS.

Twelve volatile compounds belonging to different chemical classes, monoterpenes (44.75%) and aliphatics (49.30%), were identified in the column (Figure 3A). The most abundant compounds were α-pinene, 2,6-dimethylnonane, eucalyptol, and 1,3-di-tert-butylbenzene, accounting for about 71% of the total GC peak area, followed by 2,4-dimethyl-1-decene (8.18%), *cis*-1,1,3,4-tetramethylcyclopentane (6.75%), and 7-methyl-1-undecene (2.52%).

In the labellum, sixteen volatile compounds were identified (Figure 3B): monoterpenes (48.34%), aliphatics (48.28%), and sesquiterpens (0.61%). The most abundant compounds were 4,8-dimethyl-1,3,7-nonatriene, trans-ocieme, linalool, and 1,3-di-tert-butylbenzene, accounting for about 57% of the total GC peak area, followed by β-myrcene (8.62%), eucalyptol (6.90%), and α-pinene (5.27%) (Figure 3B).

The sepals yielded eleven volatile compounds (Figure 3C): monoterpenes (82.86%), aliphatics (8.15%), and sesquiterpenes (7.81%). The most abundant compounds were linalool, *trans*-β-ocieme, and 4,8-dimethyl-1,3,7-nonatriene, accounting for about 84%, followed by β-myrcene (3.50%), α-farnesene (2.93%), and (±)-trans-nerolidol (2.25%). The relative content of linalool was significantly higher in the sepals compared to the column and labellum.

In the petals, ten volatiles were identified (Figure 3D): monoterpenes (91.17%), aliphatics (1.33%), and sesquiterpens (6.60%). The major compounds were linalool, accounting for approximately 80%, followed by *trans*-β-ocieme (6.13%), α-pinene (3.12%), and β-farnesene (2.19%). The relative linalool content was significantly higher in the petals than in the other floral organs. The petals and sepals of the new hybrid, *Cymbidium* “Sunny Bell” had floral scents composition ratio. The order of total peak areas (data not shown) was as follows: petals > sepals >> labellum > column.

21–28 floral scent compounds in the major volatile components of the flower of three *Cymbidium* varieties [3]. The volatiles mainly comprised monoterpenes, aliphatics, and sesquiterpenes and their contents exceeded 90% [3]. Our results revealed that aliphatics (48.28–49.30%) followed by monoterpenes (44.75–48.34%) of the column and labellum, are the major components in the floral organs of the “Sunny Bell” *Cymbidium* hybrid. The main components of the sepals and petals were found to be monoterpenes (82.86–91.17%), and sesquiterpenes (6.60–7.81%). α-pinene, linalool, eucalyptol, and 4,8-dimethyl-1,3,7-nonatriene were the major compounds responsible for the floral scent of this *Cymbidium* variety. Linalool is an acyclic monoterpene with tertiary alcohol functionality and is one of the major contributors to floral scents in nature. About 70% of the terpenes contributing to floral scents are attributable to linalool. This may be because monoterpenes have a lower boiling point than sesquiterpenes. Different species contain different types and quantities of floral volatile compounds. Linalool has been reported to show anti-inflammatory, antitumor, antioxidant, and antimicrobial activity [12]. α-pinene can be found in the essential oils of coniferous (pine) trees, rosemary, lavender, and turpentine, and exhibits antioxidant [13], anti-inflammatory [14], and antimicrobial activity [15]. Eucalyptol is a colorless oil as a natural compound, and is used in food preparations. Eucalyptol shows anti-inflammatory [16,17], gastroprotective [18], hepatoprotective [19,20], and antitumorogenic effects [21], and antimycotic [22,23] and antibacterial activity [24]. 4,8-Dimethyl-1,3,7-nonatriene was isolated and identified for the first time from cardamom oil [25]. Finally, the (3*S*)-(*E*)-nerolidol synthase sesquiterpenes responsible for pleasant scent emission are a good candidate for a regulatory role in releasing the important signaling molecule 4,8-dimethyl-1,3,7-nonatriene during the daytime [26].

This study demonstrated that the new *Cymbidium* hybrid, “Sunny Bell” flowers differ greatly in their volatile composition depending on the floral organs of the plant, a finding that provides important theoretical references for flower appreciation, breeding, and studies on aromatic volatile composition.

## 3. Materials and Methods

### 3.1. Plant Materials

The flowers of *Cymbidium* “Sunny Bell” were collected in the greenhouse floriculture research division, National Institute of Horticultural and Herbal Science (NIHHS). Wanju, Korea, in February 2016 and were identified by Dr. Mi-Seon Kim; a voucher specimen (F20160204-01) is deposited in the NIHHS. The inflorescence of *Cymbidium* “Sunny Bell” is a raceme that always exhibits inconsistent flowering. Approximately 20 g of raw floral material of *Cymbidium* “Sunny Bell” was collected between 09:00 a.m. and 11:00 a.m., on 4–5 February 2016. The flowers were moistened and immediately transported to the laboratory. In all experiments, the flowers were thinly and evenly sliced using a knife, such that they could be accommodated in a headspace vial (20 mL). Finally, 1.0 g of materials was weighed and allowed to stand for 30 min at ambient temperature.

### 3.2. Analysis of Volatile Components by HS-SPME-GC-MS

Divinylbenzene-carboxen-polydimethylsiloxane (DVB-CAR-PDMS) fibers with film thickness of 50/30 μm (Supelco, Bellefonte, PA, USA) were used in this assay. For each sample, the SPME device was inserted into the sealed vial by manually penetrating the silicone septum, and the fiber was exposed to the headspace of the sliced material after 30 min. The SPME fiber was exposed to each sample for 30 min at 40 °C. After extraction, the needle on the SPME manual holder was set to 0.5 cm in the GC injector. The fiber was then directly desorbed for 10 min. An Agilent 7000C GC-MS system (Agilent Technologies, Wilmington, DE, USA), with a DB-5MS column (30 m × 0.25 mm I.D. × 0.25 μm, Agilent Technologies, Wilmington, DE, USA) was used under the following conditions: MS transfer line heater 280 °C, injector temperature 250 °C, and operation in the splitless mode. Initially, the oven temperature was held at 60 °C for 5 min, then increased from 60 °C to 250 °C at 3 °C/min, and finally maintained for 5 min at 280 °C. Helium gas was used as the carrier at a flow rate of 1.0 mL/min. The Agilent 7000C mass spectrometer was operated in the electron ionization mode at 70 eV with a source temperature of 250 °C, the quadrupole was set to 150 °C, and scanning was performed from m/z 30 to 500 in the full-scan mode.

### 3.3. Data Analysis

The constituents were identified by matching their spectra with those recorded in the NIST 14 (National Institute of Standards and Technology, Gaithersburg, MD, USA) mass spectral library and with published data (NIST, http://webbook.nist.gov/chemistry/; Pubchem, http://pubchem.ncbi.nlm.nih.gov/; Flavornet, http://www.flavornet.org/; Chemspider, http://chemspider.com/). The major components were then identified by analysis of the fragmentation data in the MS spectra. In addition, the constituents were confirmed by comparing the retention indices (RI) or GC retention time (r.t.) data with those of authentic standards or published literature. The RI′s are calculated as shown in Equation (1) [27]

RI = 100 × n + [100 × (tx − tn)]/(tn + 1 − tn)(1)

where RI is the retention index of the unknown compound x, n is the number of carbon atoms of the n-alkane eluted before x, n + 1 is the number of carbon atoms of the n-alkane eluted after x, tx is the retention time of x, tn is the retention time of the n-alkane eluted before x, and, tn + 1 is the retention time of the n-alkane eluted after x. All the indices were calculated via three replicate measurements by injecting pure compounds. The compounds were measured as relative contents (%) and calculated automatically from the peak areas obtained by the total ion chromatographic (TIC) analysis, using Equation (2) [27]:

Relative contents (%) = (area under peak/total peak area) × 100%.(2)

## Figures and Tables

**Table 1:** Morphological characteristics of a new hybrid, *Cymbidium* “Sunny Bell”.

Cultivars	Flower		Plant Size	Peduncle Attitude	Fragrance	Bloom
	Color ^a^	Shape				
**Sunny Bell** **(Wongyuo F1-47)**	RP59B	incurved	small	erect	mild	winter (From Feb.)
**Silk Road** **(control)**	R56A	some spreading	medium	semi-upright	strong	winter (From Jan.)

^a^ Based on the Royal Horticultural Society (RHS, 2001) color chart.

**Table 2:** Comparative characteristics of flowers of a new hybrid, *Cymbidium* “Sunny Bell”.

Cultivars	Flower Width/Flower Length (cm)	Peduncle Length (cm)	No. of Flowers/Peduncle	No. of Peduncle	Preference ^a^
**Sunny Bell** **(Wongyuo F1-47)**	7.9 ± 0.5 ^b^/7.9 ± 0.4	67.1 ± 8.6	7.1 ± 1.2	4.4 ± 1.6	3.7 ± 0.9
**Silk Road** **(control)**	5.4 ± 1.6/4.7 ± 1.3	47.0 ± 3.5	5.8 ± 2.6	3.3 ± 1.0	3.9 ± 0.7

^a^ Preference evaluation was undertaken at the *Cymbidium* exhibition held at the National Institute of Horticultural and Herbal Science (NIHHS) in 2013. Poor (1)–Excellent (5). ^b^ All data are presented as mean ± standard deviation (n = 15).

**Table 3:** Percentage of volatile compounds identified in four different floral organs of a new hybrid, *Cymbidium* “Sunny Bell” using HS-SPME-GC-M.

Peak	Retention Indices ^a^	Compounds	Relative Content ^b^ (%) ± SD ^c^				
			Whole Flower (Sunny Bell)	Column	Labellum	Sepal	Petal
		**Monoterpens**	**54.49**	**44.75**	**48.34**	**82.86**	**92.17**
**1**	934	α-pinene	1.06 ± 0.20^x^	27.41 ± 2.52	5.27 ± 1.59		
**2**	990	β-myrcene	10.20 ± 1.63	0.94 ± 0.22	8.62 ± 0.31	3.50 ± 0.06	3.12 ± 0.14
**4**	1032	eucalyptol	0.74 ± 0.06	16.40 ± 1.77	6.90 ± 0.96		
**5**	1035	*cis*-β-ocimene	5.81 ± 0.48			1.65 ± 0.07	1.55 ± 0.15
**6**	1047	*trans*-β-ocimene	14.37 ± 0.97		13.94 ± 0.81	8.03 ± 0.10	7.13 ± 0.27
**10**	1121	linalool	24.31 ± 2.28		13.61 ± 3.30	69.68 ± 5.26	80.37 ± 0.68
		**Aliphatics**	**6.42**	**49.30**	**48.28**	**8.15**	**1.33**
**3**	1007	2,6-dimethylnonane		16.21 ± 1.20	2.89 ± 0.12		
**7**	1076	*cis*-1,1,3,4-tetramethylcyclopentane		6.75 ± 0.16	1.93 ± 0.08		
**8**	1080	2,4-dimethyl-1-decene		8.18 ± 0.24	2.43 ± 0.23		
**9**	1113	4,8-dimethyl-1,3,7-nonatriene	4.20 ± 0.37		18.77 ± 1.06	5.67 ± 4.58	
**11**	1128	allocimene A			1.72 ± 0.07	1.35 ± 0.33	
**12**	1133	3-isopropylidene-5-methyl-1,4-hexadiene					1.33 ± 0.09
**13**	1237	4,6-dimethyldodecane		1.71 ± 0.38	2.37 ± 0.06		
**14**	1245	1,3-di-*tert*-butylbenzene	2.22 ± 0.17	10.51 ± 2.07	9.74 ± 0.11		
**15**	1300	2-isopropyl-5-methyl-1-heptanol		1.48 ± 0.45	2.25 ± 0.08		
**16**	1309	7-methyl-1-undecene		2.52 ± 0.92	3.69 ± 0.07		
**17**	1318	hexyl octyl ether		1.65 ± 0.61	2.49 ± 0.10		
**23**	1574	(3*E*,7*E*)-4,8,12-Trimethyl-1,3,7,11-tridecatetraene				1.13 ± 0.10	
		**Sesquiterpenes**	**26.04**		**0.61**	**7.81**	**6.60**
**18**	1422	β-caryophyllene	11.80 ± 0.53		0.61 ± 0.07	1.56 ± 0.68	
**19**	1454	β-farnesene	0.55 ± 0.05			1.07 ± 0.15	1.03 ± 0.19
**20**	1480	β-ionone					1.56 ± 0.14
**21**	1504	α-farnesene	13.69 ± 0.74			2.93 ± 0.10	2.19 ± 0.34
**22**	1562	(±)-*trans*-nerolidol				2.25 ± 0.28	1.52 ± 0.20

^a^ Retention indices calculated against *n*-alkanes (C8–C16); ^b^ Relative contents (%) = (area under peak/total peak area) × 100. ^c^ All data are presented as mean ± standard deviation (n = 3).
